# The impact of physical activity and basal metabolic rate as mediators of years since menopause on sarcopenia in elderly women

**DOI:** 10.1515/med-2026-1390

**Published:** 2026-03-20

**Authors:** Xinying Dong, Lihan Zhou, Li Wang, Xinying Liu, Shugang Li

**Affiliations:** School of Public Health, Capital Medical University, Beijing, China; Fangzhuang Community Health Service Center, Beijing, China; School of General Practice and Continuing Education, Capital Medical University, Beijing, China

**Keywords:** sarcopenia, physical activity, basal metabolic rate, menopause

## Abstract

**Objectives:**

This study examined the mediating effects of physical activity (PA) and basal metabolic rate (BMR) on the relationship between years since menopause and sarcopenia in community-dwelling elderly women.

**Methods:**

Based on AWGS 2019 criteria, 718 women aged ≥65 were classified into sarcopenia, possible sarcopenia, and control groups. Multiple logistic regression and mediator models were used to assess associations.

**Results:**

BMR correlated with grip strength (r=0.292), ASMI (r=0.750), and years since menopause (r=−0.246). Years since menopause negatively correlated with grip strength and SPPB (r=−0.315, −0.381; p<0.05). It was a risk factor for possible sarcopenia (OR=1.05; 95 % CI: 1.03–1.08). Low BMR and medium PA (vs. high) increased sarcopenia risk (OR=0.95 and 2.72, respectively). Direct effect of years since menopause was β=−0.010 (p=0.001); total mediating effect was 0.019 (p<0.001), mainly through BMR (0.007) and PA (0.001).

**Conclusions:**

PA and BMR mediate the effect of years since menopause on sarcopenia risk. Longer duration since menopause decreases PA and BMR, elevating sarcopenia risk.

## Introduction

Decreased muscle mass and/or function is a clear manifestation of sarcopenia, which affects 10–27 % of people aged ≥60 worldwide [1]. As an age-related disease, sarcopenia seriously affects the quality of life of the elderly and places a great social burden on the age of aging [2]. It was assumed that the prevalence rate among women (17 %) was significantly higher than among men (12 %) [1]. As the life expectancy of women increases [3], the proportion of postmenopausal time in the life cycle of women increases, and the population of women is much larger than that of men [4]. Therefore, the prevention and treatment of sarcopenia in elderly women is urgent.

The numerous factors were found to influence the prevalence of sarcopenia in women. As evidenced by research findings, the increased likelihood of sarcopenia in elderly women is closely related to menopause [5]. It was reported that the appendicular skeletal muscle mass in postmenopausal women decreased by 6 % annually [6]. A study in Finland divided 1,627 women aged 47 to 55 into four groups: premenopausal, early perimenopausal, late perimenopausal, and postmenopausal. The grip strength of the four groups decreased significantly (p<0.001), indicating a statistically significant difference in muscle function between premenopausal and postmenopausal women [7]. This suggests that menopause may be a key factor in the functional decline of female skeletal muscles.

Currently, one of the main measures for the prevention and treatment of sarcopenia is the intervention of physical activity (PA). Studies have shown that impedance exercise is effective in the prevention and treatment of sarcopenia in the elderly [8], 9]. Increasing PA may improve the quality of life of perimenopausal and postmenopausal women, and reduce various symptoms during perimenopause [10]. PA and basal metabolic rate (BMR) have always been research hotspots for improving physical health and maintaining body function. One research found that BMR was influenced by a variety of factors, and BMR could be interpreted by PA and fat-free mass (FFM) in 80 % [11]. As people age, their BMR also decreases. Compared to the youth group, BMR of the elderly group decreased by 4.6 % (p=0.04) [12]. In recent years, it has been confirmed that BMR is related to muscle mass, and BMR is positively correlated with grip strength in the elderly (p<0.001) [13]. Especially in elderly women, the correlation coefficient between lower BMR and appendicular skeletal muscle mass index (ASMI) reached 0.7 [14]. The impact of the multiple correlation among increased BMR, PA and menopause on sarcopenia in elderly women is not yet clear.

Biological findings suggest plausible pathways through which PA and BMR mediate the relationship between menopause and sarcopenia. The decline in estrogen levels following menopause often leads to fatigue, mood disturbances, and joint discomfort [15]. Collectively, these symptoms diminish both the motivation and capacity for PA in women [16]. A reduction in physical activity decreases mechanical loading on skeletal muscle, which in turn suppresses key anabolic signaling pathways such as mTOR and accelerates protein degradation, ultimately resulting in disuse-induced muscle atrophy [17]. Concurrently, estrogen exerts a direct stimulatory effect on energy expenditure; its reduction directly lowers BMR [18]. A lower BMR reflects a state of diminished resting energy expenditure, potentially fostering a catabolic milieu that is unfavorable for muscle preservation [19]. This condition is frequently accompanied by impaired muscle oxidative capacity, further compromising muscle mass and function.

Previous studies have established that both PA and BMR are significant factors contributing to sarcopenia. Menopause, an inevitable physiological process, also has an association with sarcopenia in older women. However, as elderly women age, on the one hand, their years since menopause extend, and on the other hand, there are significant differences in BMR and PA among the population. The relationship among these three factors and their combined influence on sarcopenia is not yet clear. Therefore, a deeper understanding of this relationship is crucial in developing targeted interventions for older women suffering from sarcopenia.

## Research method

### Participants

In 2023, among the elderly health examination population participating in the Fangzhuang Community Health Service Center in Beijing, we asked them if they were willing to participate in this study and collected relevant examination information such as body composition, grip strength, walking speed, and questionnaires from elderly women. Exclusion criteria are ① Age less than 65 years old; ② Individuals with cognitive (language, perceptual, etc.) impairments or periods of mental illness; ③ Implantation of a pacemaker or joint replacement in the body that prevents BIA measurement; ④ Refusal to cooperate with the inspector.

### Diagnostic criteria

According to the diagnostic criteria released by the Asian Working Group for Sarcopenia (AWGS) 2019 [20], the ASMI measured by BIA is <5.7 kg/m^2^; Physical function assessment includes a 6-m walking speed of <1.0 m/s or a short physical performance battery (SPPB) score of ≤9 or 5-time chair stand time of ≤12s; Grip strength <18 kg; If ASMI decreases and physical function or grip strength decreases, it is diagnosed as sarcopenia; If grip strength decreases or sitting for too long, it may indicate possible sarcopenia.

## Research methods

### Questionnaire survey

Collect basic information of all participants using a survey questionnaire, including age, gender, pre-retirement occupation, marital status, etc. The Physiological Questionnaire for Elderly Women reviews the menstrual and reproductive history of elderly women, with years since menopause calculated as age minus menopausal age. The Mini Nutritional Assessment short-form (MNA-SF) questionnaire was collected to evaluate the nutritional status of participants [21]. The International Physical Activity Questionnaire (Short Version) evaluates the PA and sedentary time of the participants [22].

### Body composition, body function, and grip strength tests

The use of Inbody770 (Beisbes Medical Equipment Trading (Shanghai) Co, Ltd.) for body composition testing on the human body. Do not speak during the test, remove metal jewelry, and dress lightly. During the testing process, the subjects took off their shoes and socks, followed the instrument’s voice prompts for detection, and obtained their ASMI and BMR values. All measurements were conducted throughout in a quiet and warm indoor environment.

The short physical performance battery (SPPB) score is derived from three components: the ability to maintain balance for up to 10 s in three foot positions (side-by-side, semi-tandem, and tandem), the time required to complete a 3-m or 4-m walk, and the time needed to perform five repeated chair stands. For the standing balance test, scoring is based on the ability to maintain stability in each position. For the walking and chair stand tests, scoring is first determined by the ability to complete the task, and then refined based on the time taken to perform it. Each component is scored on a 0–4 scale, with the scores from all three tests summed to yield a total SPPB score ranging from 0 to 12. Higher total scores indicate better functional capacity, while lower scores reflect poorer functional performance. Using the Baseline BIMS digital grip strength meter for grip strength testing, the specific method is to use a sitting position. The subject is instructed to place their upper arm on the side of the body, bend their elbow 90°, and hold the grip. After applying force, the data on the display screen begins to refresh until the peak value no longer changes. The measurement data are recorded and repeated twice with both hands, and the mean is finally taken for analysis.

## Data analysis

Epidata3.1 records the relevant inspection results, and the questionnaire survey is completed online by Wenjuanxing. Body composition data is directly exported from Inbody770 and merged into the database based on the same ID. Quantitative variables are expressed using mean ± SD (standard deviation), and qualitative variables are expressed using frequency (percentage). Logistic regression analysis was employed to identify factors associated with different stages of sarcopenia in elderly women, with the regression model being adjusted based on prior analytical results. Multiple mediating effects were achieved using the Lavaan package of R 4.2.3 to analyze whether BMR and PA mediate the impact of the years since menopause on the occurrence of sarcopenia in elderly women.

## Ethics approval

This study involves human participants and ethical approval was obtained from the Capital Medical University (Approval number Z2023SY074). Participants gave informed consent to participate in the study before taking part.

## Results

### Characteristics of participants

In this study, 718 elderly women were included, among whom 96 were in the sarcopenia group, with a prevalence of 13.37 %, and 323 were in the possible sarcopenia group, with a prevalence of 44.99 %. Elderly women in the sarcopenia group had lower height and weight compared to both the control group and the possible sarcopenia group (p<0.05). They were older, had lower income levels, and the age of the possible sarcopenia group was higher than that of the control group (p<0.05). Additionally, the height, weight, and monthly income of the possible sarcopenia group were lower than those of the control group, with no statistically significant differences in other indicators (p<0.05) (Table 1).

**Table 1:** Description of general demographic data for elderly women.

Classification	Variables	Control	Possible sarcopenia	Sarcopenia	Total	*χ* ^*2*^/*F*	p-Value
General demographic information	Prevalence, n (%)	299 (41.64)	323 (44.99)	96 (13.37)	718		
	Age, year	70.05 ± 4.24	71.72 ± 4.81	73.09 ± 5.69	71.21 ± 4.83	18.663	<0.001
	Height, cm	158.66 ± 4.62	158.90 ± 5.30	154.94 ± 5.65	158.27 ± 5.24	23.983	<0.001
	Height, kg	62.95 ± 8.96	63.53 ± 8.56	50.47 ± 5.32	61.54 ± 9.44	97.119	<0.001
	Education, n (%)					6.899	0.141
	Junior high school and below	13 (4.35)	29 (8.98)	6 (6.25)	48 (6.69)		
	High school	173 (57.86)	192 (59.44)	58 (60.42)	423 (58.91)		
	College or above	113 (37.79)	102 (31.58)	32 (33.33)	247 (34.40)		
	Pre-retirement occupation, n (%)					8.608	0.072
	Public clerk	168 (56.19)	151 (46.75)	55 (57.29)	374 (52.09)		
	Workers and peasants	58 (19.40)	76 (23.53)	23 (23.96)	157 (21.87)		
	Others	73 (24.41)	96 (29.72)	18 (18.75)	187 (26.04)		
	Marriage, n (%)					2.327	0.312
	Married	262 (87.63)	270 (83.59)	80 (83.33)	612 (85.24)		
	Others	37 (12.37)	53 (16.41)	16 (16.67)	106 (14.76)		
	Residential situation, n (%)					0.263	0.877
	Living alone	30 (10.03)	36 (11.15)	11 (11.46)	77 (10.72)		
	Not living alone	269 (89.97)	287 (88.85)	85 (88.54)	641 (89.28)		
	Income, n (%)					3.061	0.216
	≥6,000	197 (65.89)	233 (72.14)	64 (66.67)	494 (68.80)		
	>6,000	102 (34.11)	90 (27.86)	32 (33.33)	224 (31.20)		
Chronic disease prevalence	Osteoporosis, n (%)	70 (23.41)	73 (22.60)	24 (25.00)	167 (23.26)	0.245	0.885
	Hypertension, n (%)	160 (53.51)	208 (64.40)	47 (48.96)	415 (57.80)	11.093	0.004
	Diabetes, n (%)	72 (24.08)	106 (32.82)	26 (27.08)	204 (28.41)	5.924	0.052
	Dyslipidemia, n (%)	139 (46.49)	171 (52.94)	44 (45.83)	354 (49.30)	3.121	0.21
Lifestyle habits	Smoke, n (%)	6 (2.01)	4 (1.24)	2 (2.08)	12 (1.67)	1.296	0.862
	Drink, n (%)	6 (2.01)	3 (0.93)		9 (1.25)	5.803	0.214
Body composition and function	ASMI	6.47 ± 0.60	6.42 ± 0.54	5.31 ± 0.27	6.29 ± 0.66	186.657	<0.001
	Grip strength, kg	23.32 ± 3.55	19.57 ± 5.13	18.60 ± 3.77	21.00 ± 4.78	74.458	<0.001
	Walking speed, m/s	1.16 ± 0.20	1.01 ± 0.19	1.07 ± 0.23	1.08 ± 0.21	46.269	<0.001
	SPPB	10.76 ± 1.32	8.67 ± 1.75	9.33 ± 2.50	9.63 ± 1.97	117.172	<0.001
Nutritional and metabolic characteristics	MNA-SF	13.52 ± 0.81	13.52 ± 0.81	12.48 ± 1.21	13.38 ± 0.94	58.967	<0.001
	BMR	1,244.40 ± 80.10	1,243.42 ± 81.41	1,097.66 ± 47.00	1,224.34 ± 91.74	149.413	0.000
Health behavior and metabolic indicators	PA, n (%)					11.952	0.018
	Low	252 (84.28)	250 (77.40)	67 (69.79)	569 (79.25)		
	Medium	9 (3.01)	13 (4.02)	3 (3.13)	25 (3.48)		
	High	38 (12.71)	60 (18.58)	26 (27.08)	124 (17.27)		
Female physiological condition	Abortion, time	1.04 ± 1.08	0.98 ± 0.95	0.88 ± 1.06	0.99 ± 1.02	0.938	0.392
	Induced abortion, time	0.91 ± 0.97	0.94 ± 0.95	0.83 ± 1.01	0.91 ± 0.97	0.424	0.655
	Age of first delivery	27.14 ± 2.75	27.26 ± 2.93	27.53 ± 3.25	27.25 ± 2.90	0.634	0.531
	Parturition, time	1.23 ± 0.48	1.27 ± 0.61	1.33 ± 0.74	1.26 ± 0.58	1.193	0.304
	Spontaneous labor, time	1.08 ± 0.62	1.17 ± 0.69	1.25 ± 0.82	1.14 ± 0.68	2.730	0.066
	Age of first pregnancy, year	26.86 ± 2.70	27.09 ± 2.91	27.34 ± 3.16	27.02 ± 2.86	1.132	0.323
	Pregnancy, time	2.32 ± 2.70	2.20 ± 1.13	2.20 ± 1.30	2.25 ± 1.96	0.343	0.710
	Age of menarche, year	14.35 ± 1.79	14.50 ± 1.93	14.70 ± 2.06	14.47 ± 1.89	1.296	0.274
	Menopausal age, year	49.87 ± 4.22	49.32 ± 4.45	49.72 ± 3.96	49.60 ± 4.30	1.332	0.265
	Years since menopause, year	20.17 ± 6.03	22.40 ± 6.64	23.38 ± 6.91	21.60 ± 6.54	13.505	<0.001

ASMI, appendicular skeletal muscle mass index; SPPB, short physical performance battery; MNA-SF, mini nutritional assessment short-form; BMR, basal metabolic rate; PA, physical activity. p-values were derived from univariate tests, ANOVA or Chi-square test.

### Comparison of indicators, PA, menstrual history and reproductive history related to sarcopenia in elderly women at different stages of sarcopenia

In elderly women, the ASMI, grip strength, and Walking speed of the sarcopenia group were significantly lower than those of both the control group and elderly individuals in the possible sarcopenia group, indicating poorer nutritional status and longer years since menopause. The ASMI, grip strength, Walking speed, SPPB score, and nutritional status of the possible sarcopenia group were markedly lower than those of the control group, with longer years since menopause compared to the control group (Table 1).

### Analysis of the correlation of the diagnostic factors that affect sarcopenia in elderly women

**Figure 1:**
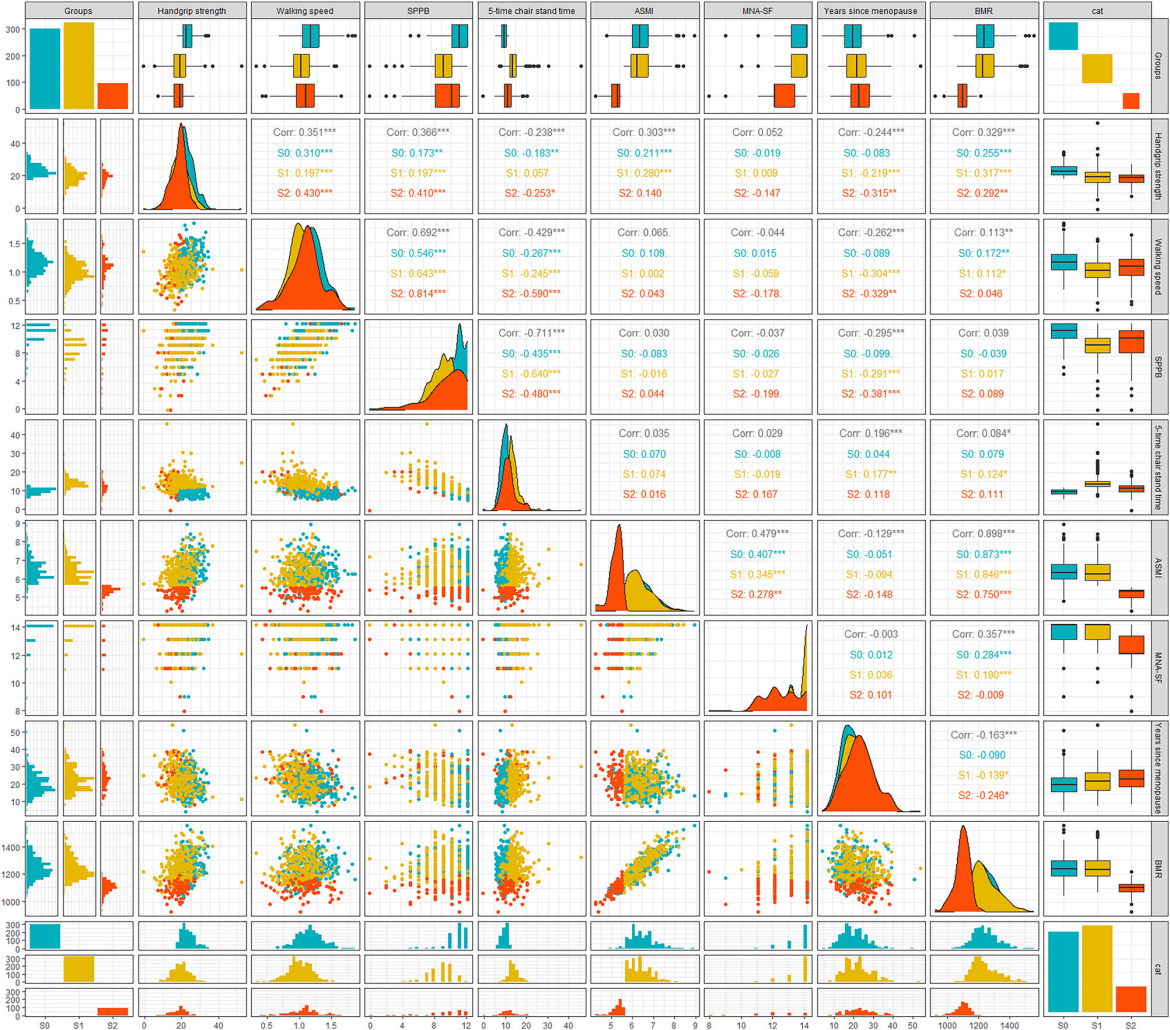
Correlation analysis between diagnostic factors of sarcopenia and BMR, PA, and years since menopause after grouping according to different stages of sarcopenia. BMR, basal metabolic rate; PA, physical activity; S0 is the control group, S1 is the possible sarcopenia group, and S3 is the sarcopenia group. ***p<0.001; **p<0.01; *p<0.05.

After categorizing all samples into the control group, possible sarcopenia group, and sarcopenia group, it was found that in the control group, BMR was correlated with grip strength, walking speed, ASMI, and MNA-SF score (r=0.255, r=0.172, r=0.873, r=0.284, respectively p<0.001). In the population of the possible sarcopenia group, correlations were observed between BMR and grip strength, walking speed, 5-time chair stand time, ASMI, MNA-SF score, and years since menopause, with correlation coefficients of 0.317, 0.112, 0.124, 0.846, 0.190, and −0.193 (respectively, p<0.05). Years since menopause were correlated with grip strength, walking speed, SPPB, and 5-time chair stand time (r=−0.219, r=0.278, r=−0.291, r=0.177, respectively p<0.001). In the sarcopenia group, BMR was correlated with grip strength (r=0.292, p<0.001), ASMI (r=0.750, p<0.001), and years since menopause (r=−0.246, p<0.05). Years since menopause was correlated with grip strength, and SPPB (r=−0.315, r=−0.381, respectively p<0.05) (Figure 1).

### Multivariate analysis of factors that affect sarcopenia in elderly women

Years since menopause is a risk factor for the possible sarcopenia population OR (95 % CI)=1.05 (1.03, 1.08) and BMR is associated with an increased risk of sarcopenia, OR (95 % CI)=0.95 (0.94, 0.96). These risk factors remained significant in the adjusted model. In Model 3, an increased years since menopause was associated with an increased risk of sarcopenia, OR (95 % CI)=1.08 (1.01, 1.16). In the original model, medium PA compared to high PA was a risk factor for sarcopenia OR (95 % CI)=2.72 (1.13, 6.51) (Table 2).

**Table 2:** Multivariate logistic analysis of sarcopenia in elderly women at different stages.

		Model 1		Model 2		Model 3	
		OR (95 % CI)	p-Value	OR (95 % CI)	p-Value	OR (95 % CI)	p-Value
Possible sarcopenia vs. control	BMR	1.00 (1.00, 1.00)	0.810	1.00 (0.99, 1.00)	0.173	1.00 (0.99, 1.00)	0.101
	Years since menopause	1.05 (1.03, 1.08)	<0.001	1.06 (1.03, 1.08)	<0.001	1.06 (1.03, 1.09)	<0.001
	High PA	Reference		Reference		Reference	
	Low PA	1.38 (0.57, 3.32)	0.474	1.37 (0.56, 3.34)	0.486	1.25 (0.51, 3.07)	0.626
	Medium PA	1.45 (0.92, 2.27)	0.108	1.41 (0.89, 2.23)	0.143	1.36 (0.86, 2.16)	0.194
Sarcopenia vs. control	BMR	0.95 (0.94, 0.96)	<0.001	0.93 (0.91, 0.95)	<0.001	0.92 (0.90, 0.94)	<0.001
	Years since menopause	1.03 (0.98, 1.09)	0.268	1.08 (1.01, 1.15)	0.024	1.08 (1.01, 1.16)	0.020
	High PA	Reference		Reference		Reference	
	Low PA	0.75 (0.10, 5.43)	0.780	0.91 (0.12, 6.73)	0.926	0.75 (0.10, 5.85)	0.781
	Medium PA	2.72 (1.13, 6.51)	0.025	2.27 (0.83, 6.24)	0.111	2.72 (0.94, 7.88)	0.065

Model 1 is the original model, Model 2 corrects for height and weight, and Model 3 corrects for height, weight, nutritional level, hypertension, and age of first pregnancy. OR, odds ratio; CI, confident interval; BMR, basal metabolic rate; PA, physical activity.

### The relationship between PA and BMR in the influence of years since menopause on sarcopenia through multiple mediation effects

**Figure 2:**
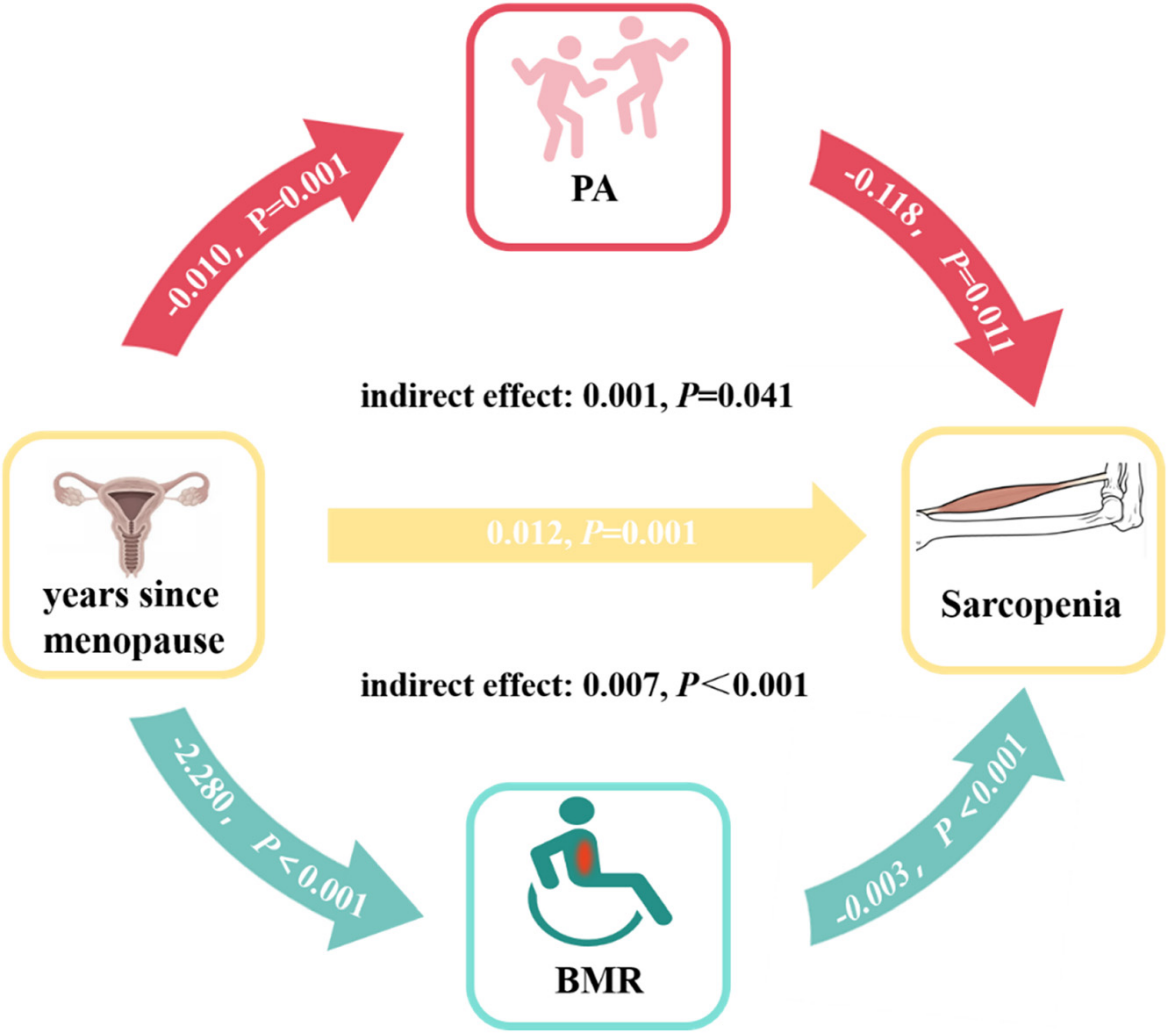
Analysis of multiple mediating effects of basal metabolic rate and PA in years since menopause and sarcopenia. BMR, basal metabolic rate; PA, physical activity.

The path coefficients and results of the mediating effect of PA and BMR in the relationship between menopausal age and sarcopenia are presented in Figure 2. Years since menopause directly influences sarcopenia, with a β coefficient of −0.010 (p=0.001). As years since menopause prolongs, both PA and BMR decrease, while the risk of sarcopenia is negatively associated with PA and BMR. The total mediating effect in the model is 0.019 (p<0.001), with indirect effects of PA and BMR on sarcopenia of 0.001 and 0.007, (respectively, p<0.05).

## Discussion

Amid the global aging process, sarcopenia significantly impacts the quality of life for many older adults and is associated with conditions such as sleep disorders and dyslipidemia [23], [24], [25], imposing a substantial disease burden. Its etiology is complex and the factors that affect older men and women are different. The appearance of menopause means that women experience cliff-like aging, which is not only related to loss of fertility but also to a decline of living ability [26]. Therefore, it is important to pay attention to the factors related to sarcopenia in elderly women. This study found that improving PA is crucial, maintaining a high BMR is an effective method to protect elderly women with prolonged menopause from sarcopenia.

In this study, the incidence of sarcopenia may be relatively high at 41.64 %, but in other communities, the incidence of sarcopenia in women may also be as high as 44.48 % [27]. In our study, we found a significant correlation between the long years since menopause and the incidence of sarcopenia. Postmenopausal women are in a low long-term estradiol state, and the decrease in estradiol is related to the release of pro-inflammatory factors. Chronic inflammatory status can reduce the proliferation and replenishment of muscle satellite cells, resulting in a significant decrease in muscle mass [28]. It is noteworthy that serum estradiol levels have been confirmed to hold significant diagnostic value for sarcopenia in perimenopausal women [29]. The deficiency of estrogen also disrupts muscle protein balance, increases protein breakdown, causes muscle fiber atrophy, and ultimately leads to loss of muscle mass and strength [30]. After menopause, the available testosterone levels in the female body also decrease, which may be one of the reasons for the accelerated loss of muscle mass in women during menopause [5].

The possible mechanism for the decline in BMR in elderly women after menopause is that estradiol (E_2_) remains at a low level for a prolonged period after menopause. E_2_ directly regulates the biophysical properties and bioenergetic functions of skeletal muscle mitochondria, low levels of E_2_ directly affect energy homeostasis, leading to a decrease in BMR [18]. The association between BMR and sarcopenia may also be attributed to decreased muscle cell renewal in the elderly, resulting in a reduction in skeletal muscle mitochondrial biogenesis and function [31]. Muscles themselves are important metabolic organs, and metabolism is the process by which energy is generated through mitochondrial oxidative reactions [32]. Thus, sarcopenia is significantly associated with BMR.

The PA of elderly women begins to decline significantly after the age of 50, and the increase in age is directly proportional to the energy expenditure required for daily activities. With aging, the energy cost of maintaining activity increases continuously for elderly individuals, leading to a decline in willingness to engage in physical activities [16]. Another reason for this phenomenon may be the increased risk of falls and osteoporosis after menopause in women, leading to an increased likelihood of fractures [33]. However, the decrease in estrogen after menopause exacerbates skin aging and prolongs wound healing, increasing the cost of recovery from fractures in elderly women [34]. Therefore, the slower wound healing rate leads to a corresponding decrease in PA in elderly women. Mitochondrial biogenesis increases with increasing PA [35], and skeletal muscle mitochondria are important organelles for meeting the energy and contraction needs of muscles [36]. Thus, the decrease in activity level is closely related to the occurrence of sarcopenia.

Recent groundbreaking advances in artificial intelligence in medicine – particularly revolutionary achievements such as AlphaFold in protein structure prediction [37] and the powerful capabilities of large language models in multimodal data processing [38] – have paved new avenues for deciphering complex disease mechanisms and enabling precision interventions. Against this backdrop, the mediating pathway of physical activity and basal metabolic rate in the relationship between postmenopausal duration and sarcopenia identified in our study provides a computable theoretical framework for developing artificial intelligence-driven health management tools.

This article has certain limitations. As a cross-sectional study conducted in the community, it cannot verify causal benefits. The BMR is estimated using the Inbody formula instead of direct measurement, and the low-activity population collected in the sample is relatively small. Therefore, the incidence of sarcopenia in the low-activity population is lower.

## Conclusions

This study found that PA and BMR play a mediating role between the years since menopause and sarcopenia. Prolonged years since menopause are associated with a decrease in PA and BMR, leading to an increased risk of sarcopenia.
